# Past, Present, and Future of Japanese Encephalitis

**DOI:** 10.3201/eid1501.080311

**Published:** 2009-01

**Authors:** Tobias E. Erlanger, Svenja Weiss, Jennifer Keiser, Jürg Utzinger, Karin Wiedenmayer

**Affiliations:** Swiss Tropical Institute, Basel, Switzerland

**Keywords:** Japanese encephalitis, emerging, rice farming, irrigation, pig rearing, vaccination program, perspective

## Abstract

Japanese encephalitis (JE), a vector-borne viral disease, is endemic to large parts of Asia and the Pacific. An estimated 3 billion people are at risk, and JE has recently spread to new territories. Vaccination programs, increased living standards, and mechanization of agriculture are key factors in the decline in the incidence of this disease in Japan and South Korea. However, transmission of JE is likely to increase in Bangladesh, Cambodia, Indonesia, Laos, Myanmar, North Korea, and Pakistan because of population growth, intensified rice farming, pig rearing, and the lack of vaccination programs and surveillance. On a global scale, however, the incidence of JE may decline as a result of large-scale vaccination programs implemented in China and India.

## Abstract 2

JE is increasing in some areas (due to population growth and intensified rice irrigation) but declining in others.

Japanese encephalitis (JE) is a vector-borne viral disease that occurs in South Asia, Southeast Asia, East Asia, and the Pacific (*1*). An estimated 3 billion persons live in countries where the JE virus is endemic (*2*), and the annual incidence of the disease is 30,000–50,000 cases (*1*). The disease can cause irreversible neurologic damage. The JE virus (JEV) is mainly transmitted by the mosquito *Culex tritaeniorrhynchus*, which prefers to breed in irrigated rice paddies. This mosquito species and members of the *Cx. gelidus* complex are zoophilic. Wading ardeid water birds (e.g., herons and egrets) serve as virus reservoirs, but the virus regularly spills over into pigs, members of the family of equidae (e.g., horses and donkeys), and humans. The annual number of human deaths is 10,000–15,000, and the estimated global impact from JE in 2002 was 709,000 disability-adjusted life years (DALYs) (*1*,*3*). However, these statistics should be interpreted with care because the transmission of JE is highly dynamic; hence, the disease usually occurs in epidemics, and there is considerable fluctuation in estimates of its global impact. In 1999, JE caused 1,046,000 DALYs; in the 2 subsequent years, it caused 426,000, and 767,000 DALYs, respectively (*3*). Underlying factors that might explain these fluctuations are contextual determinants (mainly environmental factors) and spillover effects into the human population, which trigger epidemics.

Reporting of JE cases depends on the quality of health information systems and the ability to clinically and serologically diagnose the disease. JE is often confused with other forms of encephalitis. Differential diagnosis should therefore include other encephalitides (e.g., conditions caused by other arboviruses and herpesviruses) and infections that involve the central nervous system (e.g., bacterial meningitis, tuberculosis, and cerebral malaria) (*4*).

Figure 1 shows the transmission of JE and highlights contextual determinants. Because infected pigs act as amplifying hosts, domestic pig rearing is an important risk factor in the transmission to humans (*1*). Two distinct epidemiologic patterns of JE have been described. In temperate zones, such as the northern part of the Korean peninsula, Japan, China, Nepal, and northern India, large epidemics occur in the summer months; in tropical areas of southern Vietnam, southern Thailand, Indonesia, Malaysia, the Philippines, and Sri Lanka, cases occur more sporadically and peaks are usually observed during the rainy season (*5*). Thus far, the reasons for the spread of JE are not fully understood. Bird migration might play a role in dispersing JEV (*6*). Accidental transportation of vectors, human migration, and international travel seem to be of little importance because viremia in humans is usually low and of short duration and because humans are dead-end hosts (*1*). JE was likely introduced into northern Australia by wind-blown mosquitoes from Papua New Guinea (*7*) (Figure 1).

**Figure 1:**
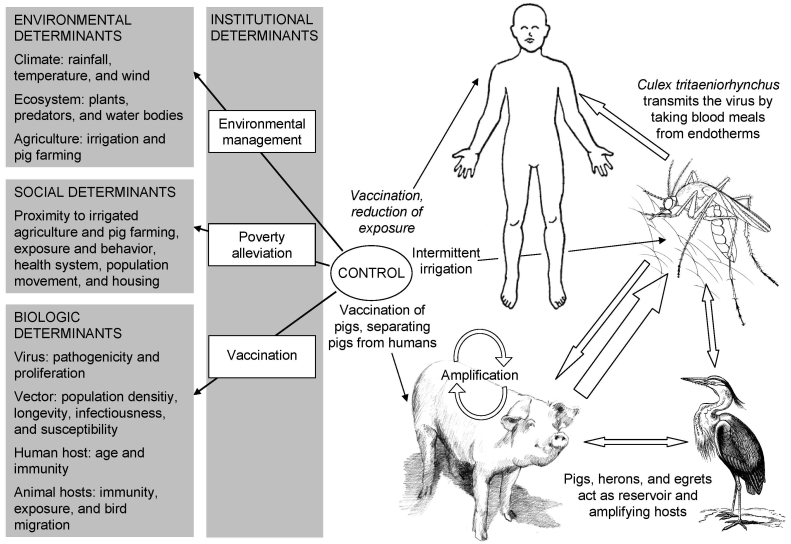
Contextual determinants and transmission of Japanese encephalitis.

The main pillar of JE control is the use of a live attenuated vaccine for humans, which was developed some 40 years ago (*8*). Currently available JE vaccines are relatively safe and effective, but a drawback is that multiple doses are required (*1*,*9*). Effective delivery of the vaccines to poor, rural communities therefore remains a formidable challenge, and compliance and delivery costs have to be considered (*10*). Two vaccine candidates are in late-stage clinical development. The first one is a second-generation, live inactivated, single-dose vaccine grown in Vero cells. It is the yellow fever virus–based chimeric vaccine and will soon enter the market (*1*). The second candidate is an attenuated SA 14–14–2 virus strain, adjuvanted with aluminum hydroxide and also grown in Vero cells (*9*,*11*).

The vaccination of pigs represents another potential strategy to control JE, but it is not widely used for 2 main reasons. First, the high turnover in pig populations would require annual vaccination of newborn pigs, which would be costly. Second, the effectiveness of live attenuated vaccines is decreased in young pigs because of maternal antibodies (*12*).

Environmental management for vector control, such as alternative wetting and drying of rice fields (also known as intermittent irrigation), can substantially reduce vector breeding while saving water, increasing rice yields, and reducing methane emission (*13*). However, an effective irrigation requires well-organized educational programs, sufficient water at specific times during the rice-growing cycle, and an adequate infrastructure. In addition, because vectors are largely dispersed, intermittent irrigation should be applied to all rice fields over large areas and during the entire cropping season, which is often not feasible (*14*). Environmental management measures are most viable if they are readily integrated into a broader approach of pest management and vector management (*15*).

Chemical control of vector populations with insecticides such as pyrethroids, organophosphates, and carbamates plays a marginal role in JE control. In some circumstances (for example, when an outbreak of JE occurs in a densely populated area), space spraying can break the transmission cycle in the short term. However, rising levels of insecticide resistance have compromised the effectiveness of this emergency measure. Indeed, JE vectors that prefer manmade habitats, such as irrigated rice fields, are often heavily exposed to pesticide selection pressure. Although JE vectors are prone to develop insecticide resistance, usually this issue arises with insecticides that are not directly targeted to JE control, but rather are targeted to control of other pests (*16*).

We provide a historic account of the origin of JE and disease epidemics, describe the current situation, and discuss several factors that might explain the rise of JE incidence in some countries and its decline in others. Finally, we speculate about possible future trends.

## Historic Account

Genetic studies suggest that JEV originated from an ancestral virus in the area of the Malay Archipelago. The virus evolved, probably several thousand years ago, into different genotypes (I–IV) and spread across Asia (*17*).

The history of the clinical recognition and recording of JE dates to the 19th century. JE appeared as recurring encephalitis outbreaks in the summer season. The first clinical case of JE was recorded in 1871 in Japan. Half a century later, also in Japan, a large JE outbreak involving >6,000 cases was documented. Subsequent outbreaks occurred in 1927, 1934, and 1935. In 1924 an agent from human brain tissue was isolated; 10 years later, it was proven to be JEV by transfection into monkey brains. The role of *Cx. tritaeniorhynchus* as a vector and the involvement of wading ardeids and pigs as reservoir hosts were demonstrated in 1938 (*18*).

Table 1 shows when the first JE cases were described in countries currently considered JE-endemic. On the Korean Peninsula, the first JE cases were recorded in 1933. On the Chinese Mainland, the first JE cases were documented in 1940. In the Philippines, first reports of JE cases occurred in the early 1950s (*19*). Eventually, the JE epidemic reached Pakistan (1983) as the furthest extension in the West, and Papua New Guinea (1995) and northern Australia (Torres Straight) as the furthest south. In parts of southeastern Russia (Primorje Promorsij), a few JE cases have been reported occasionally (e.g., 2 cases from 1986 to 1990) (*18*). JE is potentially endemic to Afghanistan, Bhutan, Brunei Darussalam, and the Maldives, but to our knowledge, no cases have been reported in these countries in the past 30 years. According to the World Health Organization (WHO), JE is endemic to the Western Pacific Islands, but cases are rare (*20*). The enzootic cycle on those Pacific islands might not sustain viral transmission; hence epidemics occur only after introduction of virus from JE-endemic areas. Subtle changes in the spatiotemporal distribution of JEV are difficult to track; thus, the year when a first case of JE in a country is reported does not necessarily correspond with the actual first occurrence of JE in that country (Table 1) (*21**–**35*).

**Table 1:** First reported case and current situation of Japanese encephalitis in the main disease-endemic countries

Country	First reported case	Total population in rural JE-endemic areas (% of total)*	Annual incidence†	DALYs in 2002‡	Trend of JE incidence§	Vaccination program†	National diagnostic center†	References
Australia	1995	NA§	<1	<1¶	Stable	Yes	Yes	(*21*)
Bangladesh	1977	106,385,000 (75)	NA	24,000	Increasing	No	No	(*22*)
Cambodia	1965#	11,293,000 (80)	NA	4,000	Increasing	No	NA	(23)
China	1940	422,532,000 (32)	8,000–10,000	281,000	Decreasing	Yes	Yes	(*18*)
India	1955	597,542,000 (54)	1,500–4,000	226,000	Increasing	No	Yes	(*24*)
Indonesia	1960	116,114,000 (52)	NA	23,000	Increasing	No	NA	(*25*)
Japan	1924	43,969,000 (34)	<10**	<1	Stable	Yes	Yes	(*26*)
North Korea	1933	8,606,000 (38)	NA	6,000	NA	NA	NA	(*27*)
South Korea	1933	9,194,000 (19)	<20	6,000	Stable	Yes	Yes	(*27*)
Laos	1989	4,643,000 (78)	NA	5,000	Increasing	No	Yes	(*28*)
Malaysia	1952††	8,854,000 (35)	50–100	2,000	Decreasing	Yes	Yes	(*29*)
Myanmar	1965	35,077,000 (69)	NA	13,000	Increasing	No	NA	(*18*)
Nepal	1978	4,567,000 (20)	1,000–3,000	5,000	Stable	Yes	Yes	(*30*)
Papua New Guinea	1995	5,109,000 (87)	NA	2,000	NA	NA	NA	(*21*)
Pakistan	1983	18,536,000 (12)	NA	82,000	Increasing	NA	NA	(*27*)
The Philippines	1950	31,081,000 (37)	10–50	8,000	Stable	No	Yes	(*19*)
Singapore	1952	0	<1	260	Stable	No	Yes	(*31*)
Sri Lanka	1968	16,381,000 (79)	100–200	1,000	Decreasing	Yes	Yes	(*32*)
Thailand	1964	43,364,000 (68)	1,500–2,500	5,000	Decreasing	Yes	Yes	(*18*,*27*)
Vietnam	1960	61,729,000 (73)	1,000–3,000	11,000	Stable	Yes	Yes	(*27*,*33*)

*Japanese encephalitis (JE)–-endemic areas drawn from map provided in (*34*); rural population estimates derived from United Nations Urbanization Revisions (*2*). †Information retrieved from questionnaires answered by employees from Ministry of Health and World Health Organization country offices. ‡Estimates for 2002 from World Health Organization (*20*). DALYs, disability-adjusted life years. §<1% of the country is JE-endemic (Torres Straight islands, Cape York Peninsula). NA, not available. ¶Two deaths since 1995 (*35*). #Virus isolated from mosquito (*29*). **JE cases are very rare; highest risk is on Okinawa, Miyako, and Ishigaki islands. Between 1995 and 2005, 3 cases were reported (all in 2002) (*26*). ††First isolated from humans. During World War II, an outbreak that was probably due to JE occurred (*31*).

## Present Situation

**Figure 2:**
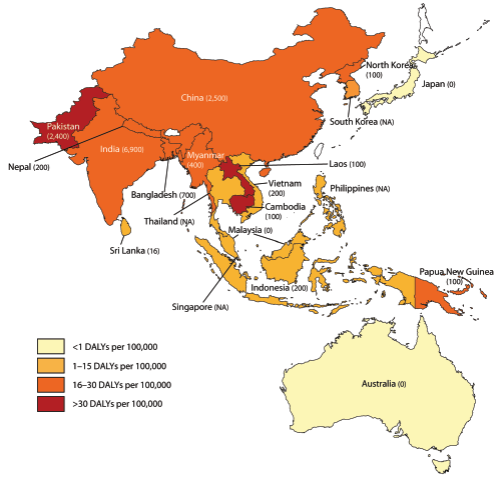
Disability-adjusted life years (DALYs) per 100,000 persons in Japanese encephalitis–endemic countries. Number in parentheses indicate estimated number of deaths in 2002 according to the World Health Organization (*20*). NA, not available.

Nearly half of the human population currently lives in countries where JEV occurs. As shown in Figure 2, JE is concentrated in China, India, and the Southeast Asian peninsula.

Current epidemiologic data on JE are summarized in Table 1. These data were gathered from a diversity of sources, including peer-reviewed literature, specialized text books, and reports from national health departments and international organizations, such as WHO and the Food and Agriculture Organization. We also contacted national health ministries and WHO country offices for up-to-date information regarding country-specific JE statistics. This was accomplished by administering a standardized questionnaire.

There are 2 distinct trends in JE incidence. In countries such as Bangladesh, Cambodia, India, and Laos, where no specific diagnostic centers, vaccination programs, and surveillance systems are in place, the incidence of JE appears to have increased in recent years. On the contrary, in China, Japan, Nepal, South Korea, Sri Lanka, and Thailand, where vaccination programs are being implemented and regular surveillance is pursued, the incidence of JE is stable or declining. Despite the availability of WHO estimates, the situation in North Korea, Myanmar, Pakistan, and Papua New Guinea remains largely unknown.

However, underreporting is substantial in most JE-endemic countries; hence, it is conceivable that annual JE incidence is considerably higher than heretofore reported. For example, an estimate that used a representative incidence of 25/10,000 (not immunized), and a 1994 population estimate of 700 million children <15 years of age who live in JE-endemic areas suggested 175,000 cases annually with 43,750 deaths, and 78,750 cases with lasting sequelae. Adjusted for vaccine coverage, the estimate is 125,000 cases per year (*36*).

## Emerging JE

The emergence of JE can probably be explained by 2 factors. First, JE-endemic countries experienced an unprecedented population growth in recent decades. For example, in Eastern Asia, South-Central Asia, and Southeast Asia, the population more than doubled, from 1.7 billion in the mid-1950s to 3.5 billion 50 years later (*2*). Second, pig rearing has grown exponentially and rice-production systems, particularly irrigated rice farming, have increased both in cropping area and cropping intensity. In China, for example, pork production doubled from 1990 to 2005. Today, the total rice-harvested area of all JE-endemic countries (excluding the Russian Federation and Australia) is 1,345,000 km^2^, an increase of 22% in the past 40 years. Over the same time span, the total rice production in these countries has risen from 226 million tons to 529 million tons (+134%) (*37*).

Table 2 presents information on 3 key environmental contextual determinants of JE transmission, stratified by country. The following issues are offered for consideration. First, the number of people living in close proximity to irrigated areas reflects the fraction of the population that potentially is at an elevated risk of acquiring JE. Methods for calculating those numbers have been described elsewhere (*13*). Second, the absolute and relative change of irrigated rice area and, third, the relative change in pork production can be used as proxies for alterations in the risk of acquiring JE. In absolute numbers (116.6 million) and in relative terms, most people living in close proximity to irrigated areas are from Bangladesh (82%); the second largest population lives in India (107.8 million), followed by China (22.0 million). The largest irrigated rice area in 2005 was found in India (41.9 million ha), followed by China (29.0 million ha), Bangladesh (10.5 million ha), and Thailand (10.0 million ha). Highest increases in irrigated rice areas in the past 15 years were estimated for Myanmar (+47%) and Cambodia (+30%). Highest increases in pork production occurred in Myanmar (+381%), Vietnam (+147%), and China (+87%). On the other hand, pork production declined in Malaysia (–47%), North Korea (–35%), and Japan (–23%).

**Table 2:** Rice irrigation and pork production by Japanese encephalitis–endemic country, 1990 and 2005*

Country	Persons in close proximity to rice irrigation†	Rice paddy area, 1990†	Rice paddy area, 2005†	% Change in paddy area	% Change in pork production†
Australia	NA	NA	NA	NA	NA
Bangladesh	116,600,000	10,435	10,524	+1	NA
Cambodia	1,426,000	1,855	2,415	+30	+46
China	22,019,000	33,519	29,087	–13	+87
India	107,785,000	42,687	41,907	–2	–8
Indonesia	7,169,000	10,502	11,802	+12	–13
Japan	1,947,000	2,074	1,706	–18	–23
North Korea	414,000	600	590	–2	–35
South Korea	921,000	1,244	980	–21	+69
Laos	164,000	NA	NA	NA	+21
Malaysia	181,000	681	676	–1	–47
Myanmar	3,120,000	4,760	7,008	+47	+381
Nepal	554,000	1,455	1,542	+6	+12
Papua New Guinea	110	NA	NA	NA	NA
Pakistan	513,000	2,113	2,621	+24	NA
The Philippines	12,200,000	3,319	4,200	+27	+18
Singapore	0	0	0	0	NA
Sri Lanka	2,232,000	828	915	+10	+21
Thailand	8,330,000	8,792	10,042	+14	+80
Vietnam	18,648,000	6,043	7,329	+21	+147

*NA, not available. †Sources of data and methodology of calculation are described by Keiser et al. (*13*). ‡Source: Food and Agriculture Organization (*37*).

Despite the fact that irrigated rice production and pig rearing are key factors in the transmission of JE, crude numbers fail to completely explain the complex interplay of various contextual determinants of the disease. Clearly, where rice production and pig rearing overlap, the impact on JE transmission is stronger than in areas where both activities are physically separated. This is the case, for example, in Malaysia, where the Malays mainly grow rice in 1 area and the Chinese rear pigs in another area. Here, the social determinant of religion (most Malays are Muslim) plays a decisive factor (Table 2).

## Conclusion and Outlook

Discovered 125 years ago, JE has spread widely in the 20th century. Almost half of the human population now lives in countries where the disease is endemic. JE is a vector-borne epidemic with several features that are typical of an emerging infectious disease. The failure to halt the spread of JE in Asia and the Pacific region, despite the availability of an effective and inexpensive vaccine for 40 years, is of considerable public health concern. A similar conclusion has been drawn for yellow fever, a disease that can also be prevented by vaccination yet is rampant (*38*). Similar to schistosomiasis, malaria, food-borne trematodiasis, lymphatic filariasis, and dengue, one of the main reasons for the proliferation of JE is the ecologic transformation caused by water resources development and management that create suitable breeding sites for vectors and intermediate hosts, which in turn influence the frequency and transmission dynamics of these diseases (*39*).

High-quality data on transmission and incidence of JE are lacking in various countries. Although clinical and serologic methods to diagnose and monitor JE are available, health systems in many developing countries are unable to differentiate encephalitis diagnoses. Information regarding the distribution and public health importance of JE in Bangladesh, Cambodia, Indonesia, North Korea, Laos, Myanmar, Papua New Guinea, and Pakistan is inadequate. Such epidemiologic information, however, is mandatory for advocacy and allocation of resources for the control of JE. Examples of countries with successful JE control programs are Japan and South Korea. Before the 1950s, these countries experienced JE outbreaks, but incidence rates have remained stable for >2 decades. The following key control strategies and developments might explain the successful decline of JE in these countries: 1) large-scale immunization programs for humans, 2) pig immunization and the separation of pig rearing from human settlements, 3) changes in agricultural practices (e.g., enhanced mechanization and decrease of irrigated land), and 4) improved living standards (e.g., better housing and urbanization).

We speculate that JE incidence is increasing mainly in low-income countries. However, because reliable figures about JE emergence are lacking due to the absence of rigorous monitoring systems, more research is needed to support or refute this claim. In any event, lack of political will and financial resources are 2 important reasons why JE is often given low priority. These factors might explain the paucity of JE immunization programs for children in low-income countries where the disease is endemic. Nevertheless, Sri Lanka and Nepal, 2 countries with limited health budgets, and Thailand and Vietnam have managed to successfully control JE.

The national situations with respect to JE in the near future could develop as follows. We hypothesize that in Cambodia, Laos, and Myanmar, severe JE outbreaks could occur in the near future, partially explained by increases in irrigated rice farming and enhanced pig rearing. The JE situation in North Korea is not well understood, but on the basis of the population’s general health status, we predict that JE will likely remain a substantial public health issue in the years to come. Bangladesh and Pakistan are among the worst affected and most populous countries in which JE is endemic, and yet effective surveillance is missing. Outbreaks are likely to occur but will remain largely undetected. Muslim countries such as Bangladesh and Pakistan have traditionally been JE free. JEV transmission ends in Pakistan, even though the JE vector is abundant further to the West. The recent rise in JE in those countries has yet to be fully investigated and shows the complexity of transmission of this disease. In Indonesia, Malaysia, the Philippines, and Singapore, JE incidence has usually been low, and transmission will remain stable at a relatively low level. Given the paucity of data in Indonesia, a monitoring system should be established to document changes over time. Occasional small JE outbreaks might also occur in Papua New Guinea with spillover to Australia. Awareness of the disease and vaccination coverage rates are high in Australia, particularly in the region of the Torres Strait; hence, it seems unlikely that larger epidemics will occur anytime soon.

The overall trend of JE has been declining over the past 3 decades, and we anticipate that this trend will continue in the long term. Indeed, China and India influence JE figures on a global scale because most people living in JE-endemic areas are concentrated in these 2 countries. The incidence of JE in China has declined since 1971, coincident with economic growth and development. Meanwhile, the national JE vaccination program has been integrated into the Expanded Program on Immunization, and, at present, >110 million doses of a live, attenuated vaccine (SA14–14–2 strain) are produced annually. However, social, economic, and health policy changes in the face of privatization and a more market-based economy have led to reduced funding for immunization programs and somewhat reduced salaries for public health workers, particularly in the poorest provinces. As a consequence, these changes have contributed to increasing disparities in immunization coverage rates between the wealthy coastal and the less developed rural provinces and thus to the recently observed differences in levels of JE incidence between those regions (*40*).

The incidence of JE in India is still increasing, and the case-fatality rate of reported cases is high, i.e., 10%–30% (Technical Appendix, supplementary reference 41). India currently has no national vaccination program, but the Ministry of Health has recently drawn up a plan in which children 1–12 years of age will be immunized. In Tamil Nadu and Uttar Pradesh, immunization programs are already running; thus, JE incidence might stabilize in those regions. However, overall trends for India are difficult to predict because JE endemicity is heterogeneous and because socioeconomic conditions for control differ substantially from 1 state to another (Technical Appendix, supplementary reference 42).

Coverage of immunization programs and changes in agricultural practices will further influence JE transmission. In Taiwan, for example, the average age for the onset of confirmed JE cases shifted from children <10 years toward adulthood, explained by a high coverage of vaccinated children (Technical Appendix, supplementary reference 43). Interestingly, the peak JE transmission, which occurred in August in the 1960s, shifted to June beginning in the 1980s. Improvements in pig-feeding technologies, which resulted in shorter periods from birth to pregnancy of female pigs, has been proposed as an important reason explaining the shift in transmission (Technical Appendix, supplementary reference 44).

Climate change has been implicated in the increase of transmission of several vector-borne diseases (Technical Appendix, supplementary reference 45). For example, a potential effect of climate change has been shown empirically for dengue virus, which is closely related to that of JE (Technical Appendix, supplementary reference 46). Although JE vector proliferation might be influenced in a similar way than that predicted for dengue vectors, the potential impact of climate change on JE remains to be investigated. Indeed, climate change could not only directly increase JE vector proliferation and longevity but could also indirectly increase disease because of changing patterns of agricultural practices such as irrigation (Technical Appendix, supplementary references 47,48). Areas with irrigated rice-production systems may become more arid in the future, and the impact of flooding will be more dramatic, which in turn might result in JE outbreaks. Generally, extreme rainfall after a period of drought can trigger outbreaks in situations in which vector populations rapidly proliferate and blood feeding is spilling over to humans. Climate change may also influence migration patterns of birds, which may result in JEVs being introduced into new areas. However, little is known about reservoir bird migration patterns; hence, this issue remains to be investigated (*6*).

The culicines that transmit JE are usually highly zoophilic, and human outbreaks are therefore the result of a spillover of the virus from the animal reservoir into the human population. Studies in Sri Lanka showed that spillovers happen when there is rapid and dramatic buildup of *Culex* spp. populations to the extent that the number of human blood meals passes a threshold after which virus transmission begins (Technical Appendix, supplementary reference 49). Such rapid buildups are a result of extreme weather conditions or of rice fields in semi-arid areas being flooded before rice is transplanted. Information on vector population dynamics would be very useful in early warning systems and could also help improve targeting of control programs.

In conclusion, JE can be controlled, with effective surveillance systems and vaccines playing key roles. Although currently available vaccines are effective, the need for 3–4 injections compromises compliance and increases delivery costs (*10*). The advent of second-generation, cell-culture–derived vaccines will continuously replace mouse-brain and hamster kidney cell–derived vaccines. Such developments will hopefully boost current vaccination programs and deliver safer, more efficacious, and cheaper vaccines that comply with regulatory norms. Political will and commitment, financial resources, intersectoral collaboration (between the Ministries of Health and Agriculture and other stakeholders to set up vaccination programs for young children), as well as changing agricultural practices, pig vaccination, rigorous monitoring, and surveillance will go a long way in controlling JE.

## Supplementary Material

Technical Appendix Past, Present, and Future of Japanese Encephalitis
