# Supplementary material for: Past, Present, and Future of Japanese Encephalitis
Source: Emerg Infect Dis. 2009 Jan;15(1):1–7. doi: 10.3201/eid1501.080311 (PMC2660690; doi:10.3201/eid1501.080311)
Supplement: Technical Appendix — Past, Present, and Future of Japanese Encephalitis [file 08-0311_Techapp-s1.pdf]

Past, Present, and Future of Japanese Encephalitis

Technical Appendix

Supplementary References 41–49

41. Parida M, Dash PK, Tripathi NK, Ambuj, Sannarangaiah S, Saxena P, et al. Japanese encephalitis outbreak, India, 2005. Emerg Infect Dis. 2006;12:1427–30. Medline

42. Jacobson J, Sivalenka S. Japanese encephalitis globally and in India. Indian J Public Health. 2004;48:49–56. Medline

43. Wu YC, Huang YS, Chien LJ, Lin TL, Yueh YY, Tseng WL, et al. The epidemiology of Japanese encephalitis on Taiwan during 1966–1997. Am J Trop Med Hyg. 1999;61:78–84. Medline

44. Tseng HF, Tan HF, Chang CK, Huang WL, Ho WC. Seroepidemiology study of Japanese encephalitis neutralizing antibodies in southern Taiwan: a comparative study between urban city and country townships. Am J Infect Control. 2003;31:435–40. Medline DOI: 10.1067/mic.2003.73

45. Khasnis AA, Nettleman MD. Global warming and infectious disease. Arch Med Res. 2005;36:689–96. Medline DOI: 10.1016/j.arcmed.2005.03.041

46. Hales S, de Wet N, Maindonald J, Woodward A. Potential effect of population and climate changes on global distribution of dengue fever: an empirical model. Lancet. 2002;360:830–4. Medline DOI: 10.1016/S0140-6736(02)09964-6

47. Hsu SM, Yen AM, Chen TH. The impact of climate on Japanese encephalitis. Epidemiol Infect. 2008;136:980–7. Medline DOI: 10.1017/S0950268807009454

48. Yasuoka J, Levins R. Ecology of vector mosquitoes in Sri Lanka–suggestions for future mosquito control in rice ecosystems. Southeast Asian J Trop Med Public Health. 2007;38:646–57. Medline

49. Peiris JS, Amerasinghe FP, Amerasinghe PH, Ratnayake CB, Karunaratne SH, Tsai TF. Japanese encephalitis in Sri Lanka–the study of an epidemic: vector incrimination, porcine infection and human disease. Trans R Soc Trop Med Hyg. 1992;86:307–13. Medline DOI: 10.1016/0035-9203(92)90325-7
